# Chemokines and Glycosaminoglycans

**DOI:** 10.3389/fimmu.2015.00246

**Published:** 2015-05-26

**Authors:** Amanda E. I. Proudfoot

**Affiliations:** ^1^NovImmune, Geneva, Switzerland

**Keywords:** chemokines, glycosamininoglycans, RANTES, GAG mutants, oligomerization

Although it was not known at the time, the chemokine field started with the identification of a protein by Heparin Sepharose affinity chromatography, platelet factor 4 (PF4) (1) now called CXCL4 in the systemic nomenclature introduced in 2000. As with many pathways in scientific research, our entry into the field of chemokines and glycosaminoglycans (GAGs) was a series of fortuitous co-incidences. Christine Power, working on the chemokine project in the Glaxo Institute of Molecular Biology (GIMB) under the leadership of Tim Wells, had hired a postdoctoral scientist, Arlene Hoogewerf to clone murine CCR4, and to generate the KO mouse. Arlene had previously studied the role of proteoglycans in regulating the function of other molecules and had also previously published a paper on the enzymatic activity of CTAP-III, and NAP-2, truncated forms of platelet basic protein/CXCL7, to degrade heparin. Since our group was currently working on these chemokines derived from the β-thromboglobulin precursor, and moreover we were all biochemists, our interest was piqued by the relationship between chemokines and GAGs. This interest was of course inspired by the paper recently published by Antal Rot demonstrating the evidence for haptotaxis as opposed to chemotaxis (2), earning him the title of Godfather of Chemokine–GAG biology. Arlene then followed a dual Post-doc pathway, partially hijacked by Tim to collaborate with him and his Ph.D. student, Gaby Kuschert (now Gaby Campanella) to lay much of the biochemical groundwork of chemokine–GAG interactions, and also fulfilling her goal of creating the CCR4^−/−^ mouse. They were able to show that chemokines demonstrated selectivity in their interaction with GAGs, beyond the obvious electrostatic interactions between basic and acidic molecules, and importantly made the observation that this interaction could trigger oligomerization of chemokines (3, 4). In addition, they were able to define the pharmacophore responsible for GAG binding of the chemokine IL-8/CXCL8 (5).

My lab became more directly involved in this research direction through the serendipitous encounter of a Ph.D. student, Sarah Fritchley, working in Simi Ali’s lab in Newcastle, UK, who was interested in expressing the putative GAG binding mutant of RANTES/CCL5 in *E. coli*, but who did not have a viable expression system. Sarah spent a couple of months in the lab under the tuition of our expert chemokine protein chemist, Fred Borlat, successfully producing the 40’s mutant, as we colloquially called it. We published it in JBC with its correct biochemical nomenclature, ^44^AANA^47^-RANTES (6). Perhaps an omen as to the importance of this mutant for us was that it was an exception among most papers we had submitted as it was accepted overnight!

We were heavily involved in screening for chemokine receptor antagonists at this time, and Marie Kosco-Vilbois had set up a simple cell recruitment assay to test putative inhibitors *in vivo –* chemokine-induced peritoneal recruitment in mice. Therefore, to investigate the effect of the abrogation of GAG binding *in vivo*, we asked her technician, Suzanne Herren, to test it for us. Being very rigorous, and accustomed to testing compounds for their ability to inhibit chemokine induced recruitment, in this instance RANTES, Suzanne tested it both for its agonist and antagonist activity. I will never forget my amazement in seeing that the mutant was not only unable to recruit cells but actually inhibited the recruitment induced by RANTES.

Again serendipity stepped in. I gave a talk at a BALR meeting in the UK, and after dinner at the speaker’s table, joined the youngsters at the adjacent table for a post-prandial “relaxation”… (which would never happen in this day and age). There I met Zoë Johnson, whom I learned the next day, was toying with the idea of doing a Ph.D., after having worked for a few years as an *in vivo* pharmacology laboratory assistant in *in vivo* pharmacology in industry. Since I was responsible for the Student program at the Institute, now the Serono Pharmaceutical Research Institute, she emailed me soon after expressing her interest. Tim was by now Director of the Institute, but had kept his interest in the chemokine project close to his heart, and having the flair and ability to make quick opportunistic decisions, immediately approved her appointment to investigate this phenomenon further.

We then embarked on a marvelous and exciting 3 years of research during Zoë’s thesis as the importance of the chemokine–GAG interaction unveiled itself. This period was also the beginning of my collaboration with Tracy Handel, one of the most enjoyable and mutually fruitful collaborations between two laboratories that I have had the pleasure to be part of, where we shared everything without any trace of competiveness, leading to several “duo” presentations of our joint discoveries at chemokine meetings. The result of these 3 years, during which we had access to other GAG binding mutants, notably those of MCP-1/CCL2 produced by Tracy’s lab in Berkeley, CA, USA, and that of MIB-1β from Patti Liwang in Texas, was the demonstration that the immobilization of chemokines on GAGs was essential for their *in vivo* activity, and that moreover, certain chemokines needed to form oligomers in order to exert their property of cellular recruitment *in vivo* (7). The inter-relationship between these two properties was shown by the failure to include another chemokine–GAG mutant, that of murine MIP-1α, sent to us by Gerry Graham in Glasgow. He included of course the WT control, which in our hands was inactive in recruiting cells *in vivo*, despite his assurance that it was fully active *in vitro*, so we saw no use in testing his GAG binding mutant in our in *in vivo* assay. What he neglected to tell us was that in line with the work carried out by Lloyd Czapeklski at British Biotech some years previously the WT chemokine had been mutated to no longer oligomerize – obviously an obligate monomer that was inactive *in vivo*, in accordance with our results with the three obligate monomers described above!

We then exploited the inter-relationship of GAG binding and oligomerization using our RANTES mutant, now commonly called 004, in our lab, an abbreviation of its company nomenclature used for all biologicals, AS900004, and AANA-RANTES to our collaborators, as a very effective anti-inflammatory tool (8–10). However, the fact that it retained agonist activity precluded its development as a biological therapeutic. However, we felt that we were on the right path to discovering a novel set of molecules that would interfere with the chemokine–GAG interaction and would give us a superior niche to differentiate from our competitors who were all targeting the chemokine:receptor interaction. To achieve this, we used two approaches. The first lead by our talented head of chemistry, Matthias Schwarz, was to carry out an approach coined “SAR by NMR” to identify protein binders. Our target protein RANTES/CCL5 had the advantage that (a) it was small and therefore amenable to NMR technology, and (b) its three dimensional structure in complex with a GAG – a disaccharide – had been solved by our X-ray crystallographer, Jeffrey Shaw (11). The aim was to screen a small library of about 200 sulfated compounds by NMR to identify RANTES/CCL5 binders. The first screen yielded a hit, which prevented binding to heparin, and inhibited RANTES-induced peritoneal cell recruitment, despite only having micromolar affinity. The aim was to then identify a second molecule in a second round of screening, this time in the presence of the first compound, and then using the data obtained from the structures of the complexes solved by Jeff, to design a linker to form a dimer, which would have considerably higher affinity. The screening and structural biology arms worked beautifully, and the dimer was synthesized by the chemists – but the product no longer inhibited cell recruitment *in vivo* – and much to our chagrin and despair, it even enhanced it.

However, we were still believers and decided to follow another lead. Zoë had shown that the minimal repeating unit of heparin that could inhibit RANTES-induced peritoneal recruitment was a tetrasaccharide. We therefore hired another postdoctoral scientist, India Severin, a chemist whose objective was to identify and then synthesize GAG-based mimetics. Despite a very assiduous program in collaboration with a glycobiology group in Australia, led by Deidre Coombe, we had to admit defeat. Although we identified moieties that inhibited GAG binding to RANTES as well as RANTES binding to the receptor CCR1, we did not achieve our aim of identifying a lead candidate for an anti-inflammatory program (12).

To my delight, several years later, Deidre contacted me with the explanation as to why the design of our dimer resulting from our screen by NMR was incorrect. We had performed our crystallization studies at an acidic pH in order to maintain the monomeric form of RANTES, which would crystallize without aggregating. Modeling studies at physiological pH values revealed that our compounds had bound to the protein at acidic pH in a manner different from that predicted by the docking studies at physiological pH, presumably due to their different protonation states (13). And even more consoling was the publication of a GAG moiety that had anti-inflammatory properties in a model of lung inflammation by preventing T-cell recruitment (14).

However, we still have a long way to go to fully understand the inter-relationship between the two interactions that chemokines have, especially *in vivo*. Chemokine biologists have always talked about gradients, but without defining whether these gradients are in the fluid phase or caused by immobilized chemokines through their interaction with GAGs. Our work showed that chemokines needed to be immobilized but did not address the question of a gradient. This has recently been beautifully demonstrated by Michael Sixt, where he visualized gradients of CCL21 leading to lymphatic vessels (15). We believed that the active form of the chemokine must be that, which is immobilized on the extracellular surface. However, our recent work at Novimmune, with Nicolas Fischer and Marie Kosco-Vilbois and another very talented Ph.D. student, Pauline Bonvin, characterizing two anti-murine CXCL10 antibodies, has led to revisiting this hypothesis. The mAb that is active in *in vivo* models of disease does not recognize GAG bound chemokine, whereas the mAb that is ineffective does, a result, which contradicts the notion that it is the GAG bound form of the chemokine that is active *in vivo* (Bonvin et al., manuscript in preparation). However, the active mAb inhibits the binding of the chemokine to GAGs, indicating that this interaction does indeed play a role, but the point of intervention appears more subtle that initially thought. Hopefully, more detailed studies of these two antibodies will provide a greater in depth understanding of the role of GAG binding in chemokine-induced cell migration *in vivo*.

## Conflict of Interest Statement

The author declares that the research was conducted in the absence of any commercial or financial relationships that could be construed as a potential conflict of interest.
